# The potential influence of high uric acid exposure on surface and corrosion susceptibility of pure titanium

**DOI:** 10.1007/s10856-022-06667-2

**Published:** 2022-05-16

**Authors:** Yao Liu, Wen-si Zhang, Ze-hua Tang, Song-mei Zhang, Jing Qiu

**Affiliations:** 1 Department of Oral Implantology, Affiliated Hospital of Stomatology, Nanjing Medical University, Nanjing, China; 2 Jiangsu Province Key Laboratory of Oral Diseases, Nanjing Medical University, Nanjing, China; 3 Department of General Dentistry, Eastman Institute for Oral Health, University of Rochester, Rochester, NY USA; 4 Jiangsu Province Engineering Research Center of Stomatological Translational Medicine, Nanjing, China

## Abstract

This study investigated the corrosion susceptibility of pure titanium under uric acid exposure for 7 days based on surface analysis. The prepared pure titanium specimens, exposed to different concentrations of uric acid, were examined for surface microstructure, surface element composition and surface wettability using scanning electron microscopy (SEM), X-ray photoelectron spectroscopy (XPS) and static contact angle measurement, respectively. The corrosion behaviors of titanium specimens were measured by open-circuit potential (OCP), electrochemical impedance spectroscopy (EIS) and potentiodynamic polarization. The titanium ion release from the prepared specimens, which were immersed in Hank’s balanced salt solution (HBSS) containing different amount of uric acid, was measured by inductively coupled plasma atomic emission spectrometry (ICP-AES). More irregular pitting holes were observed on titanium surfaces exposed to a high concentration of uric acid, and XPS analyses revealed that the amount of titanium dioxide (TiO_2_) decreased. Titanium surfaces pre-treated with high uric acid became more hydrophobic. Furthermore, the results of OCP and potentiodynamic polarization tests showed increased corrosion susceptibility of titanium samples, while EIS data indicated more active corrosion behavior of titanium materials. The high concentration of uric acid also induced titanium ion release. High concentration of uric acid negatively influenced the surface characteristics and corrosion properties of titanium materials, which destroyed the titanium oxide film barrier. High uric acid exposure increased corrosion susceptibility of pure titanium specimens and accelerated titanium ion release.

**Figure:**
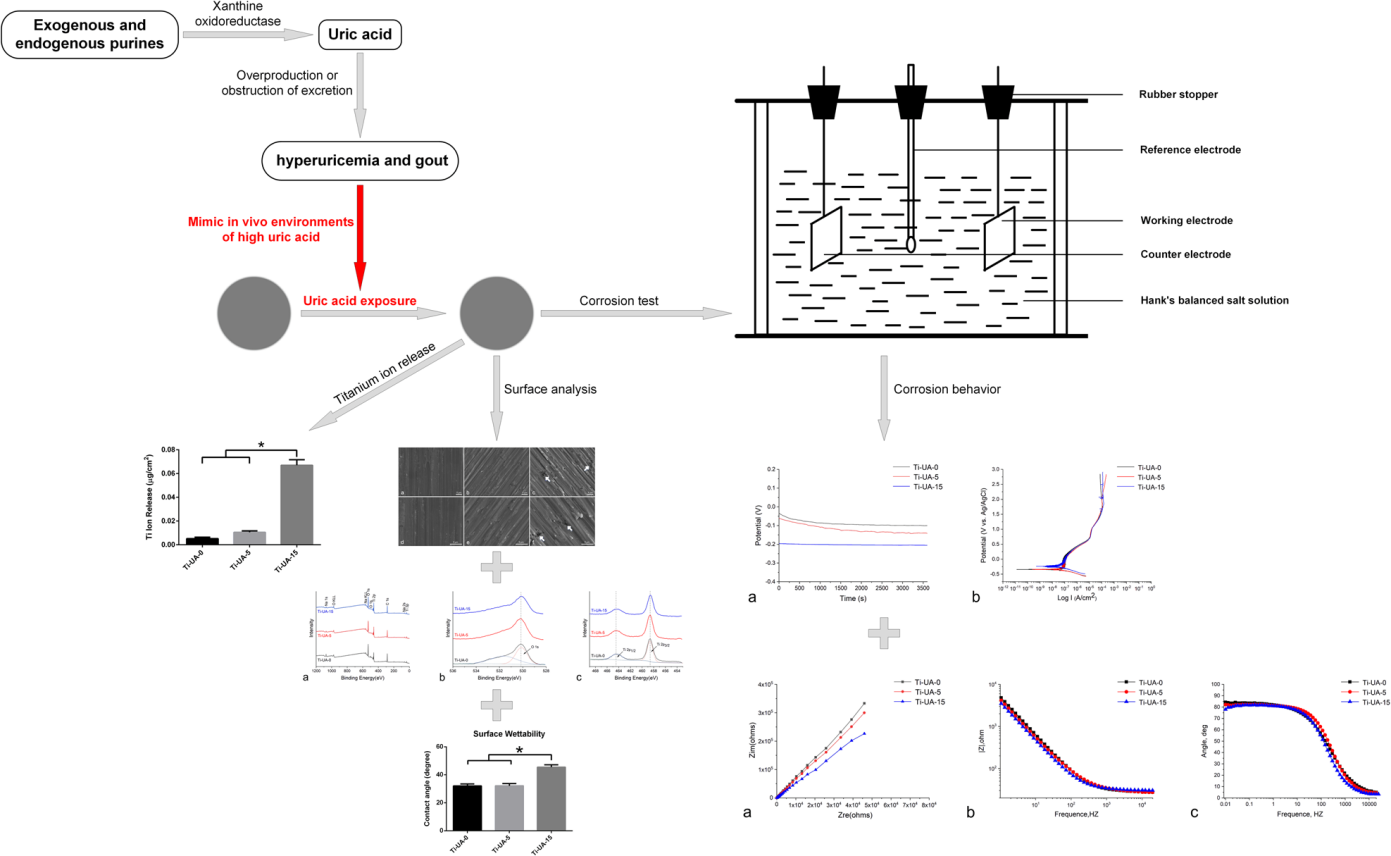
Graphical abstract

Graphical abstract

## Introduction

Titanium and its alloys have seen extensive use in orthopedic and dental implants due to their remarkable mechanical properties, chemical stability and biocompatibility [1]. Many modification methods have been utilized on titanium surfaces to improve oral implant performance [2–4]. Titanium materials possess better biocompatibility and corrosion resistance than other implant materials, however, corrosion caused by exposure to some aggressive agents, such as bodily fluids, remains a concern [5]. Upon insertion into the jaw, blood-rich tissue surrounds the titanium implants, and certain substances present in the blood directly impact the corrosion susceptibility of implant materials.

After completing the dental implantation, blood infiltration occurs during the first contact with the implant surface [6, 7]. Furthermore, some substances in the blood, such as metabolites, may also affect the surface characteristics of implant materials and change the composition of the surrounding environment, which causes an electrochemical reaction and the subsequent corrosion process [8]. Therefore, the corrosion behavior of titanium materials upon biological fluid contact likely explains the deterioration of its surface properties and subsequent cellular responses.

Uric acid is a weak organic acid produced when purine metabolizes. Xanthine, the immediate precursor of uric acid, is degraded into uric acid by xanthine oxidoreductase. Additionally, the primary sources of uric acid and xanthine are both exogenous and endogenous purines in the human body [9]. In patients with hyperuricemia and gout, the plasma concentration of uric acid tends to be excessive. The occurrence of hyperuricemia and gout has increased lately due to the improved quality of life and altered diet structures [10–12]. The relationship between hyperuricemia and gout has been extensively investigated [12, 13]. Furthermore, hyperuricemia appears to be a potential contributor to metabolic disorders, hypertension, kidney diseases and cardiovascular disease [14]. Many studies have shown that some substances in bodily fluids play a critical role in altering the corrosion behavior of implant materials [15–19]. However, whether elevated levels of uric acid in patients with hyperuricemia and gout make titanium implants more prone to corrosion has not been reported.

During recent years, it has been well established that the electrochemical corrosion testing was regarded to be superior to the traditional immersion testing to characterize the corrosion properties of metallic biomaterials [20–22]. Corrosion tests play a significant role in metallic biomaterial research since higher corrosion rates lead to the release of more metal ions. Lower corrosion rates are desirable because corrosion interferes with cell metabolisms in the surrounding tissue and may result in the implant failure [23, 24]. Nevertheless, a systematic study concerning the effect of uric acid exposure on the surface characteristics and corrosion properties of titanium materials has not been published.

To gain more insight, we conducted a comprehensive study to evaluate the effects of uric acid on pure titanium materials, including their surface microstructure, surface chemical composition, surface wettability, electrochemical corrosion properties and metal ion release. This research hypothesized that high uric acid breaks down the oxide films on titanium surfaces and increases its corrosion susceptibility. Consequently, the main objective of this in vitro study was to evaluate the corrosion behavior of titanium pre-treated with different concentrations of uric acid solutions by employing open-circuit potential versus time (E versus t), electrochemical impedance spectroscopy (EIS) and potentiodynamic polarization.

## Materials and methods

### Specimen preparation

The specimens used in this study were commercially pure titanium (cp-Ti, China). All specimens were polished with waterproof silicon carbide (SiC) abrasive paper (600, 800, 1000, 1200 and 1500 grit). Afterward, the specimens were sonicated in distilled water, ethanol and distilled water for 15 min in succession, and then dried at room temperature for 2 h. Subsequently, samples were sterilized in an autoclave for 2 h and dried for 24 h in an oven at 65 °C.

### Specimen treatment with uric acid

Initially, 2 g of NaOH was dissolved in 100 mL distilled water to form a 0.5 M NaOH solution. Then 150 mg uric acid (Ourchem, China) was added to the NaOH solution, swirling until fully dissolved. The concentration of the uric acid solution was 15 mg/mL and stored at room temperature. Before the experiments, the 15 mg/mL uric acid solution was diluted to the final concentration of 0 mg/dL、5 mg/dL (normal uric acid concentration) and 15 mg/dL (high uric acid concentration) using Hank’s balanced salt solution (HBSS). Subsequently, 2 mL different concentrations of uric acid (0 mg/dL、5 mg/dL and 15 mg/dL) were added to the titanium specimens in 24-well plates, respectively. The titanium specimens immersed in 0 mg/dL and 5 mg/dL uric acid solutions were denoted as Ti-UA-0 and Ti-UA-5, respectively, and used as the control groups. Those immersed in 15 mg/dL of uric acid were denoted as Ti-UA-15 and used as the experimental group. Titanium specimens pre-treated with different concentrations of uric acid were incubated at 37 °C in a humidified 5% CO_2_ environment for 7 days. Afterward, the specimens were sonicated orderly in distilled water, ethanol and distilled water for 15 min, followed by drying at room temperature.

### Titanium surface characterization

The surface microstructure of prepared specimens was investigated by field emission scanning electron microscopy (SEM, 1530VP, LEO, Germany). The X-ray photoelectron spectroscopy (XPS, Thermo Scientific Escalab 250Xi, USA) was employed to characterize the surface chemical composition and the chemical states of each sample utilizing a monochromatic Al Kα electrode (15 kV, 150 W and 45° take-off angle). Survey and high-resolution spectra were acquired using pass energies of 160 and 40 eV, respectively. Reference binding energy for each element was obtained from the National Institute of Standards and Technology XPS Online Database (http://srdata.nist.gov/xps/). Spectra were calibrated by adjusting the binding energy of C 1 s to 284.8 eV. The surface wettability of titanium samples was evaluated from the static contact angle measurement of a 5 µL droplet of deionized water onto the substrates by using an Automatic Contact Angle Meter Model SL200B (Kenuo, USA) at room temperature. All measurements were performed in triplicate.

### Electrochemical corrosion test

Before testing, five specimens from each group after uric acid exposure were carefully mounted in a self-cured epoxy resin, exposing 0.785 cm^2^ surfaces, and sonicated using distilled water, anhydrous ethanol and distilled water for approximately 5 min. Corrosion tests were conducted using an electrochemical potentiostat (CS310H, Wuhan Corrtest Instrument Co., Ltd) through a test cell with the mounted specimens as the working electrode, a high-purity platinum wire as the counter electrode, and Ag/AgCl as the reference electrode. Corrosion tests were performed in triplicate for each specimen in HBSS at 37 ± 0.5 °C.

Each specimen remained in a steady open circuit potential (OCP) for an hour, followed by the application of a 10 mV amplitude sine wave potential through a frequency range of 1000 kHz to 10 mHz. Electrochemical impedance spectroscopy (EIS) tests were implemented using the dedicated Power Sine software. The acquired data, including Nyquist plot, Bode |Z| and Bode Phase angle diagrams, were analyzed and fitted using an appropriate equivalent circuit using the ZsimpWin software. The Ecorr was recorded and then a potentiodynamic polarization test was initiated within a scanning range from −400 to +3000 mV (versus reference electrode) at a sweep rate of 1 mV/s. The curvetting routine of the dedicated PowerSuite software (CS Studio 5, Wuhan Corrtest Instrument Co., Ltd) was applied to analyze the acquired polarization curves to calculate the corrosion current (Icorr) and the polarization resistance (R_p_) of the titanium materials.

### Measurement of titanium ion release

Immersion tests were employed to evaluate the amount of titanium ions released from different specimens. Three specimens were prepared in each group and immersed in different concentrations of uric acid solution. Specimens were incubated in a humidified environment containing 5% CO_2_ for 7 days at 37 °C. Subsequently, the Specimens were removed and the soak solutions were analyzed for titanium ion release by inductively coupled plasma atomic emission spectrometry (ICP-AES Vista AX, Varian Inc., Palo Alto, CA, USA), using matrix-matched standards. Those results, in ppm, were converted to μg ∙ cm^−2^ of titanium surface area. All measurements were performed in triplicate.

### Statistical analysis

Data were statistically analyzed using the software program IBM SPSS Statistics (v22.0; IBM Corp) via one-way analysis of variance (ANOVA). *P* < 0.05 was set as the threshold for statistical significance.

## Results

### Surface microstructure observation

**Fig. 1:**
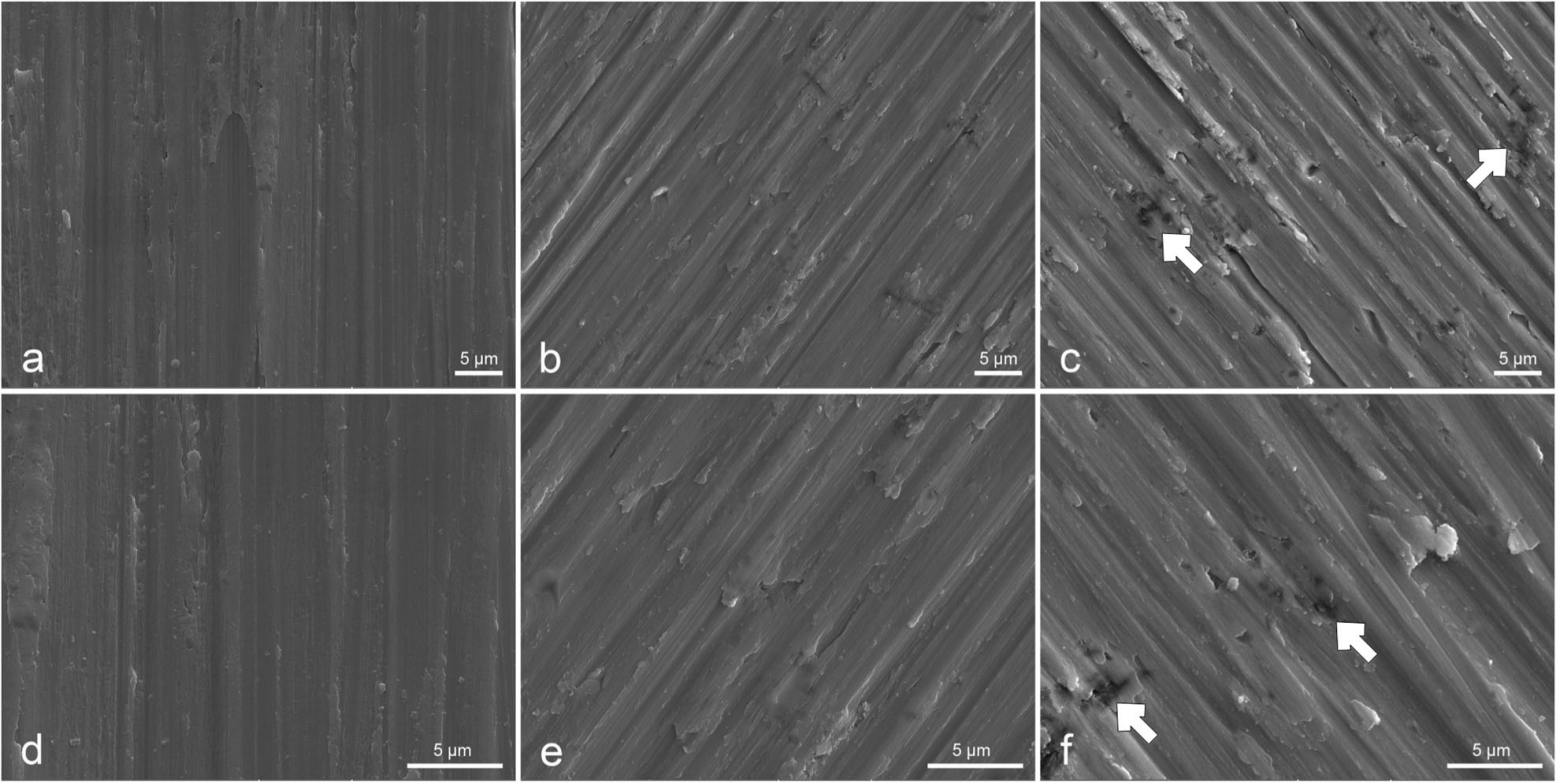
SEM images of titanium samples under different concentrations of uric acid exposure for 7 days. Upper panel ((**a**)Ti-UA-0; (**b**)Ti-UA-5; (**c**)Ti-UA-15) images at 5000 × magnification. Lower panel ((**d**) Ti-UA-0; (**e**) Ti-UA-5; (**f**) Ti-UA-15) images at 10,000 × magnification

Figure 1 shows the SEM microstructure images of the three samples. After sonicating three times, no obvious uric acid crystals were detected on Ti-UA-0, Ti-UA-5 and Ti-UA-15 samples. The surface morphologies of Ti-UA-0 and Ti-UA-5 samples did not show obvious differences under 5000× and 10,000× magnifications (Fig. 1a, b, d and e). However, different from the surfaces of Ti-UA-0 and Ti-UA-5 samples, some irregular pits and relatively rough surfaces were observed on the Ti-UA-15 samples (Fig. 1c, f).

### Analysis of X-ray photoelectron spectroscopy

**Fig. 2:**
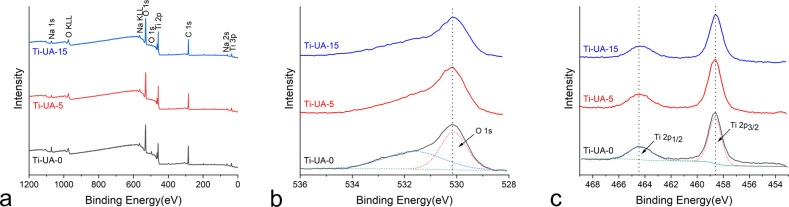
XPS spectra of different titanium samples ((**a**) survey spectra of the samples; (**b**) high-resolution spectra of O 1 s on the samples; (**c**) high-resolution spectra of Ti 2p on the samples)

XPS survey spectra obtained from titanium surfaces pre-treated with 0, 5 and 15 mg/dL uric acid are displayed in Fig. 2a. After the specimens were treated with different concentrations of uric acid for 7 days, oxygen (O), titanium (Ti), carbon (C) and sodium (Na) were detected on the Ti-UA-0, Ti-UA-5 and Ti-UA-15 surfaces. The O 1 s and Ti 2p peaks of the Ti-UA-15 group showed a declining trend compared with the two control groups. The adventitious carbon (C) peak was probably attributed to contamination, while the sodium (Na) most likely derived from the HBSS solution. XPS high-resolution spectra of O 1 s and Ti 2p on different titanium surfaces are displayed in Fig. 2b, c. From XPS high-resolution spectra analyses, the O 1 s peak was attributed to the peak at 530.1 eV, which corresponds to the titanium oxide (Fig. 2b). The Ti 2p peaks for titanium specimens in the Ti-UA-15 group (Fig. 2c) were attributed at 464.6 eV (TiO_2_) and 458.5 eV (TiO_2_), which indicated the presence of the TiO_2_. Our results revealed a reduction in the amount of both oxygen and titanium dioxide on the titanium surfaces pretreated with 15 mg/dL of uric acid solution. These findings implied that a high concentration of uric acid pre-treatment destroyed the oxide film and surface microstructure of the titanium specimens.

### Surface wettability measurement

**Fig. 3:**
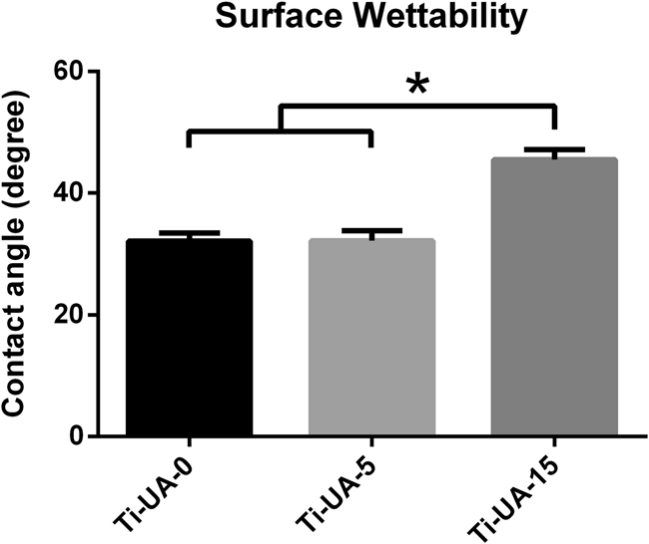
Contact angles of different titanium surfaces pre-treated with 0 mg/dL、5 mg/dL and 15 mg/dL uric acid. Results are presented as mean ± SD (*p < 0.05)

Figure 3 shows the water contact angles of Ti-UA-0, Ti-UA-5 and Ti-UA-15 surfaces. The titanium surfaces of the two control groups showed a relatively low contact angle of about 31°. After treatment with 15 mg/dL uric acid for 7 days, the titanium surfaces became more hydrophobic relative to the controls.

### Corrosion behavior

**Fig. 4:**
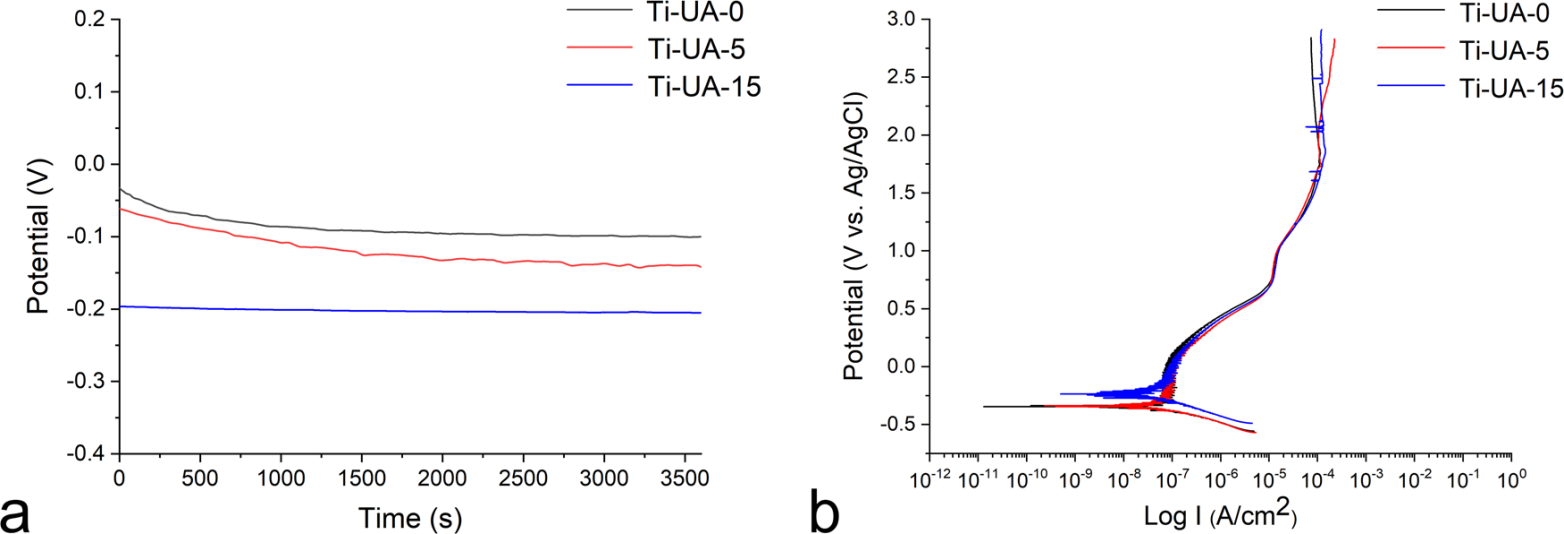
Representative OCP curve and typical diagram of the potentiodynamic polarization for different titanium samples. **a** OCP curve; (**b**) potentiodynamic polarization curve

**Fig. 5:**
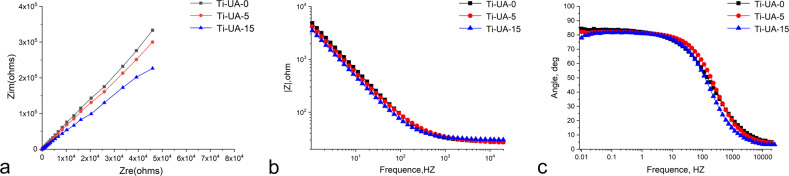
Electrochemical impedance spectroscopy of pure titanium specimens in HBSS with different uric acid concentrations. **a** Nyquist plot diagram for different titanium samples; (**b**) Bode |Z | diagrams for different titanium samples; (**c**) Bode phase angle diagrams for different titanium samples

Figures 4 and 5 illustrate the results of open circuit potential (OCP), electrochemical impedance spectroscopy (EIS) and potentiodynamic polarization for titanium specimens pretreated with 0, 5 and 15 mg/dL uric acid solutions. Representative OCP curve and typical diagram of the potentiodynamic polarization for Ti-UA-0, Ti-UA-5 and Ti-UA-15 groups are displayed in Fig. 4a, b. The OCP values of cp-Ti decreased after high uric acid exposure (Fig. 4a). The polarization tests (V vs. Log I, Fig. 4b) showed that the cp-Ti surface became much more susceptible to corrosion attack after the treatment of 15 mg/dL uric acid. Figure 5a features the Nyquist plot diagrams for different titanium specimens. There were no obvious differences in the Ti-UA-0 and Ti-UA-5 groups. However, the diameters of the impedance loops decreased when the specimens pretreated with 15 mg/dL uric acid solution compared to the controls. Representative electrochemical impedance data, including Bode |Z| and Bode Phase angle diagrams, are displayed in Fig. 5b, c. Those results showed a drop in the low-frequency impedance (based on Randle’s circuit approximation of the interface, associated with the oxide resistance) and a narrowing of the phase angle frequency range with high uric acid exposure. The decrease in impedance modulus at the lowest frequency manifested the corrosive effect of high uric acid on pure titanium surfaces. As shown in Fig. 5c, the pure titanium specimens immersed in 0 and 5 mg/dL uric acid solutions, showed phase angles close to 84° and 82° at 0.01 Hz. After immersion in solution with 15 mg/dL uric acid solution, the phase angles of cp-Ti specimens declined at the lowest frequency of 0.01 Hz. The results from EIS data analysis showed that the corrosion behavior of pure titanium surfaces agreed with the open circuit potential (OCP) and potentiodynamic polarization results.

The spectra of Ti-UA-0, Ti-UA-5 and Ti-UA-15 samples were interpreted with an equivalent circuit model of R_**s**_ (R_p_Q), which are typical for the passive oxide layer. In this model, R_s_ represents the electrolyte resistance, R_p_ represents the corrosion resistance of the surface oxide layer, which is inversely proportional to corrosion rate, and Q represents the constant phase elements (CPE) of the inter-barrier layer. The CPE, including Y_0_ and n, represents a shift from ideal capacitive behavior. The corresponding values of R_p_, Y_0_-CPE, n and χ^2^ are listed in Table 1. The chi-square values were ~10^−3^, indicating that the experimental data were consistent with the fitted values. The experimental group demonstrated a significantly lower R_p_ value (*P* < 0.05) than the control groups.

**Table 1:** Corrosion parameter values for studied cp-Ti exposed to HBSS with different concentrations of uric acid.

Metal	Condition	Impedance Parameters (*n* = 3)			
		R_p_	Y_0_-CPE	*n*	χ^2^
cp-Ti	HBSS with 0 mg/dL UA	28.99 (1.91)	4.12E-5	0.90	10^−3^
cp-Ti	HBSS with 5 mg/dL UA	28.14 (1.03)	3.68E-5	0.90	10^−3^
cp-Ti	HBSS with 15 mg/dL UA	23.93 (0.96)	5.18E-5	0.89	10^−3^
ANOVA		*P* < 0.05*	–	–	–

Values: Mean (standard deviation); R_p_ (MΩ∙cm^−2^); Y_0_-CPE (μF∙cm^−2^). * indicates a statistical difference (*P* < 0.05) of corrosion parameter values among different groups

### Titanium ion release

**Fig. 6:**
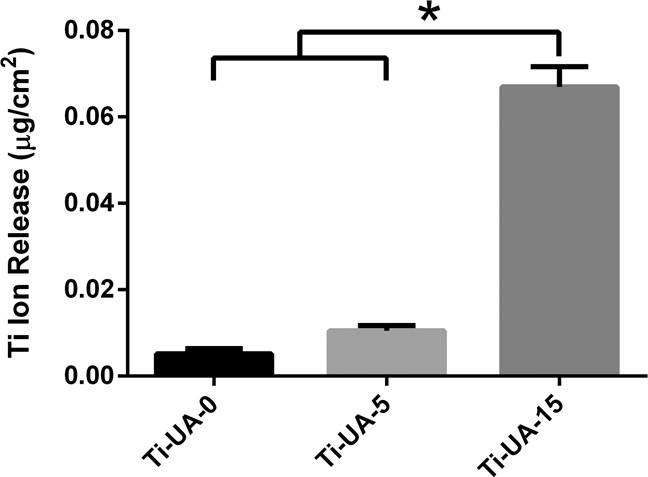
Total amount of titanium ions released from different samples pre-treated with 0 mg/dL, 5 mg/dL and 15 mg/dL uric acid solution. Results are presented as mean ± SD (*p < 0.05)

Figure 6 shows the number of titanium ions released into different concentrations of uric acid solution over a 7-day immersion period. After immersing in the 15 mg/dL uric acid solution, titanium ion release from the specimens increased significantly, while there was no obvious difference between the Ti-UA-0 and Ti-UA-5 samples.

## Discussion

Few studies have investigated the influence of uric acid on the surface characteristics and corrosion properties of titanium materials that depend on surface oxides to resist corrosion once dental implantation is finished. Previous studies demonstrated that metabolic byproducts, such as urea and ammonia, do impact the corrosion behaviors of diverse nonprecious dental alloys and pure titanium [25, 26]. Uric acid is a product of purine metabolism, and the concentration of uric acid in plasma tends to be excessive in patients with hyperuricemia and gout [13].

This study evaluated the effects of uric acid pretreatment on the surface characteristics, corrosion properties and ions releasee of pure titanium materials. The SEM, XPS and corrosion test data showed that an elevated concentration of uric acid (15 mg/dL) destroyed the protective oxidation film of titanium surfaces, weakened the corrosion resistance of titanium materials, and resulted in titanium ion release. Furthermore, the titanium surfaces became more hydrophobic with high concentration of uric acid pre-treatment. These findings supported our research hypothesis.

SEM results indicated more small pitting holes on the titanium surfaces after treatment with 15 mg/dL of uric acid solution for 7 days in the Ti-UA-15 group than in the Ti-UA-0 and Ti-UA-5 groups (Fig. 1). The white arrows on the samples under SEM represent the initiation sites of pitting corrosion, which is a form of corrosion observed mainly in passive metals such as titanium. The formation of pits without reestablishment of the oxide layer due to oxygen inhibition characterizes the corrosion [27]. These results verified that high levels of uric acid altered the surface microstructure of pure titanium materials and resulted in poor corrosion resistance. Regarding the destroyed oxide layers on the surfaces of pure titanium materials, the relative elemental amounts and their chemical states within the range of 10 nm of the surface were analyzed by XPS (Fig. 2) [5, 28]. The main component of the oxide film on pure titanium surfaces was TiO_2_, and the XPS data showed that the relative amounts of oxygen (O) and titanium (Ti) on the titanium surfaces decreased relative to the Ti-UA-0 and Ti-UA-5 groups, which meant the titanium oxide film was broken [29]. The destroyed oxide film on titanium surfaces led to increased corrosion susceptibility [20, 22]. For a better understanding of the effects of uric acid exposure on titanium surfaces, we further evaluated the surface wettability of the titanium samples over the 7-day immersion period in different concentrations of uric acid solution. As shown in Fig. 3, high uric acid exposure influenced the hydrophilicity of the titanium surfaces. Combined with SEM and XPS analyses, the hydrophilicity decrease may be caused by the variation of surface morphology and elementary composition of titanium specimens pretreated with 15 mg/dL of uric acid.

Previous results have shown that metal oxides on alloy surfaces form spontaneously after polishing due to rapid oxygen adsorption from the atmosphere [30]; this oxide formation could be destroyed by substances in body fluids upon the implantation completion [15, 16, 18, 31, 32]. Uric acid, which is a product of purine metabolism, coordinates with some divalent and trivalent metal ions as a bidentate ligand through the protonated N-7 in the imidazole ring and O-6 in the pyrimidine ring [33]. Furthermore, as a chelator of transitional metal ions [34–36], high concentrations of uric acid may break down the surface oxide coating and alter the corrosion susceptibility of the alloy.

Together with the SEM observations, XPS and WCA analyses, high concentration of uric acid destroyed the oxide layer of the titanium specimens in the Ti-UA-15 group. Once pitting holes appeared on the pure titanium surfaces, the fresh titanium substrates interacted directly with electrolytes in bodily fluids to form an original battery device and produce electrochemical corrosion.

The oxide layer of titanium surfaces has been investigated extensively and plays a significant role in corrosion [37–41]. Moreover, the oxide film is considered to be a nonconductive barrier or resistor to the electron flow between the titanium and electrolyte [42]. Our results indicated that a high concentration of uric acid (15 mg/dL) destroyed the surface oxide layer, which resulted in the weakened corrosion resistance of the pure titanium. Therefore, we further investigated the corrosion behavior of titanium pre-treated with different concentrations of uric acid solution in HBSS by means of open-circuit potential versus time (E versus t), electrochemical impedance spectroscopy (EIS) and potentiodynamic polarization.

In our study, the corrosion susceptibility of pure titanium surfaces, as determined through open-circuit potential versus time (E versus t) and potentiodynamic polarization, increased in the Ti-UA-15 group, compared with the Ti-UA-0 and Ti-UA-5 groups (Fig. 4). Corrosion experiments were conducted by EIS, as this method characterizes the corrosion of titanium oxide film in a nondestructive way [43–45]. Previous studies have shown that the diameters of the impedance loops are correlated positively with corrosion resistance [43]. In the Nyquist plots (Fig. 5a), the diameters of the impedance loops decreased significantly upon exposure to the 15 mg/dL of uric acid solution, which indicated that the chemical reactivity and corrosion rate of titanium samples increased.

The Bode |Z| and Bode phase angle diagrams (Fig. 5b, c) set forth clearly the properties of the oxide layers on the studied pure titanium surfaces. As shown in Fig. 5b, c, the high phase angle and impedance magnitude appeared at the lowest frequency for titanium specimens in control groups, indicating that there was no active pit growth. The same results were obtained from SEM images (Fig. 1), which showed the smooth titanium surfaces with no obvious pitting holes after immersion in 0 and 5 mg/dL uric acid solution for 7 days. These results illustrated that low concentration of uric acid solution did not negatively influence the corrosion behavior of pure titanium materials. A higher phase shift at a lower frequency in Bode phase plots indicates a good passive film [44, 45]. Consequently, the passive film formed on titanium surfaces became visibly defective or unstable upon immersion in the 15 mg/dL uric acid solution. The Bode phase plot results were also supported by the lower R_p_ value of 0.96 after the titanium specimen immersion in the 15 mg/dL uric acid solution for 7 days. This was also corroborated by corrosion results from other studies in which titanium materials showed statistically lower R_p_ values and unstable passive film on their surfaces [44, 46].

After immersion for 7 days, the titanium specimens from the control groups released fewer titanium ions than the experimental group (Fig. 6). The release of excessive titanium ions into the surrounding tissue may cause adverse reactions [47–49]. Together with the above findings, it could be confirmed that 15 mg/dL uric acid solution triggered the release of titanium ions during the pretreatment process, which further confirmed the XPS and corrosion test results. Consequently, elevated uric acid could be considered to reduce the corrosion resistance of titanium materials.

It is well established that the application of titanium materials has achieved a high success rate in medical fields, such as orthopedic and dental implants, with few serious short-term and long-term clinical sequela. However, as we learn more about titanium materials, electrochemical corrosion processes may compromise the clinical outcome of titanium implants and lead to some adverse effects [50, 51]. Our research primarily evaluated the influence of uric acid on the surface characteristics and corrosion susceptibility of titanium materials. We hope the results of this study lay a solid foundation for the elucidation of the exact effects of uric acid exposure on the titanium implants and the subsequent cellular response.

## Conclusions

In this study, we investigated the effects of uric acid exposure on the surface characteristics and corrosion susceptibility of titanium materials. The surface micromorphology, elemental composition and surface wettability of titanium materials changed under high uric acid exposure. Moreover, this study confirmed that high uric acid exposure influenced the corrosion susceptibility of titanium materials. We discussed a possible mechanism of uric acid exposure on titanium corrosion behavior—as a chelator of transitional metal ions, the high concentration of uric acid may destroy the surface microstructure and titanium oxide film. High uric acid would reduce the corrosion resistance and accelerate the release of titanium ions from pure titanium materials. The results of this study provide evidence for the improvement of dental implant treatment modes in patients with hyperuricemia and gout.

## Supplementary information

Editorial Certificate

## Data Availability

The datasets used or analyzed during the current study are available from the corresponding author on reasonable request.
